# Analgesic effect of premixed nitrous oxide in postoperative rehabilitation for ankle fractures: a randomized controlled trial

**DOI:** 10.1080/07853890.2026.2685317

**Published:** 2026-06-10

**Authors:** Xueling Qiu, Fei Wang, Yuchen Wang, Jiayi Dou, Wenjuan Cao, Liting Zhang, Chenxi Sun, Weifeng Wang, Lu Tang

**Affiliations:** ^a^ Department of Stomatology, The 960th Hospital of People’s Liberation Army of China (PLA), Jinan, China; ^b^ School of Nursing, Shandong First Medical University & Shandong Academy of Medical Sciences, Tai’an, China; ^c^ Department of Anesthesiology, The 960th Hospital of People’s Liberation Army of China (PLA), Jinan, China; ^d^ School of Nursing, Jinzhou Medical University, Jinzhou, China; ^e^ School of Nursing, Shandong Second Medical University, Weifang, China; ^f^ School of Nursing, Shandong University of Traditional Chinese Medicine (TCM), Jinan, China

**Keywords:** Ankle fractures, postoperative rehabilitation, nitrous oxide, pain

## Abstract

**Introduction:**

The global incidence of ankle fractures is on the rise, effective postoperative rehabilitation is a crucial aspect of surgical management. However, the occurrence of severe pain during the rehabilitation continues to pose a substantial clinical challenge.

**Methods:**

The study utilizes a dual-arm, single-center, double-blind, randomized controlled trial design. A total of 100 participants were enrolled. Participants included patients experiencing acute pain (self-reported pain score ≥4) who underwent postoperative rehabilitation for ankle fractures. Participants undergoing rehabilitation training were randomized to receive either 65% nitrous oxide or 100% oxygen. The primary outcome measured was the pain score, while secondary outcomes encompassed anxiety scores, physiological indices, side effects, patient and therapist satisfaction, acceptance, and residual pain.

**Results:**

Pain scores were found to be significantly lower in the nitrous oxide group than in the oxygen group (T1: median difference −3.0, [95% CI −3.4 to −2.6]; *p* < 0.001; T2: median difference −2.0, [95% CI −2.8 to −1.2]; *p* < 0.001). Compared with the oxygen group, both therapists (*p* < 0.001) and participants (*p* < 0.001) in the nitrous oxide group reported significantly higher satisfaction levels. Acceptance rate showed significant intergroup differences (*p* < 0.001). No severe adverse effects were observed in either group. Anxiety scores between the two groups did not show a significant difference (*p* = 0.31, η^2^ = 0.027).

**Conclusion:**

The safety and analgesic profile of nitrous oxide renders it a suitable option for pain management in postoperative ankle fractures rehabilitation, thus providing a safe and easily manageable alternative to the current available strategies.

**Clinical trial registration:**

We have registered at https://www.chictr.org.cn and the registration number is: ChiCTR2400089379.

## KEY MESSAGES

- In comparison with oxygen gas, nitrous oxide has been demonstrated to reduce pain during rehabilitation training following ankle fracture surgery, offering a practical and effective alternative to oral analgesics.
- The use of nitrous oxide in postoperative rehabilitation training for ankle fractures is relatively safe, with few and mild adverse reactions.
- Patients and rehabilitation therapists reported high satisfaction with nitrous oxide intervention, and residual pain was significantly reduced.

## Introduction

Ankle fractures are one of the most common lower limb fractures [1]. There is a growing body of evidence to suggest that incidence rates have increased over the past few decades [2]. A study reported that the incidence of ankle fractures ranged from 42 to 187 per 100,000 people [3,4]. While the majority of fractures can be treated non-operatively, unstable ankle fractures typically require operative reduction and internal fixation (ORIF) as the primary treatment [5]. The conventional postoperative protocol involves six weeks of below-knee cast or boot ankle immobilization and non-weight-bearing [6,7]. Although immobilization of the ankle can provide support and protection for the fracture site during the early stages of healing, this also increases the risk of stiffness and residual pain [1].

Rehabilitation treatment is used to address the sequelae of ankle fractures and immobilization. The rehabilitation process following ankle fractures is a complex one, comprising a range of techniques and exercises designed to facilitate ankle mobility, muscle strength, weight-bearing and balance control [8]. Nevertheless, patients commonly report significant discomfort and pain during the rehabilitation process. Such difficulties may be attributed to complications, including prolonged immobilization, soft tissue adhesion, and other complications. Such factors have been demonstrated to inhibit the recovery and movement of the injured limb, thus diminishing the effectiveness of joint rehabilitation [9].

Currently, there is no evidence-based clinical analgesic protocol that is optimal for use during rehabilitation exercises. Oral drugs are usually the main treatment methods for immediate to short-term postoperative pain [10,11]. Common medications include opioids and NSAIDs [12,13]. Despite their capacity to alleviate pain, these medications are associated with a range of adverse effects [11]. Oral drugs take a certain amount of time to take effect before achieving effective analgesia; therefore, they need to be prescribed in advance by the clinician and taken by the patient in advance to be effective. In addition, medications are administered only when the patient experiences increased and intolerable pain. In most cases, the therapist will not recommend medication during recovery, and patients are usually treated without analgesics.

Nitrous oxide (N_2_O) is a short-acting inhalant that has been demonstrated to have anaesthetic, analgesic and anxiety-relieving effects [10]. It is virtually insoluble in blood, does not bind to haemoglobin, has a rapid response, is metabolized by the lungs after cessation of inhalation, and has few side effects [14]. This method is effective, rapid, reliable, and non-intrusive, and has been shown to alleviate discomfort in patients. In recent years, it has been employed in a variety of contexts, including oral clinic, painless delivery, postoperative dressing changes and emergency treatment [15–18]. It has a wide range of clinical applications and significant analgesic effects. Given these characteristics, N_2_O seems to be an appropriate analgesic for rehabilitation exercises following ankle fractures.

The aim of this study was to examine the analgesic impact of the N_2_O inhalation technique in the context of postoperative rehabilitation for ankle fractures. We hypothesized that the use of N_2_O would be effective in reducing pain reported by participants during postoperative ankle fractures rehabilitation with mild side effects.

## Methods

### Study design and participants

**Figure 1.**
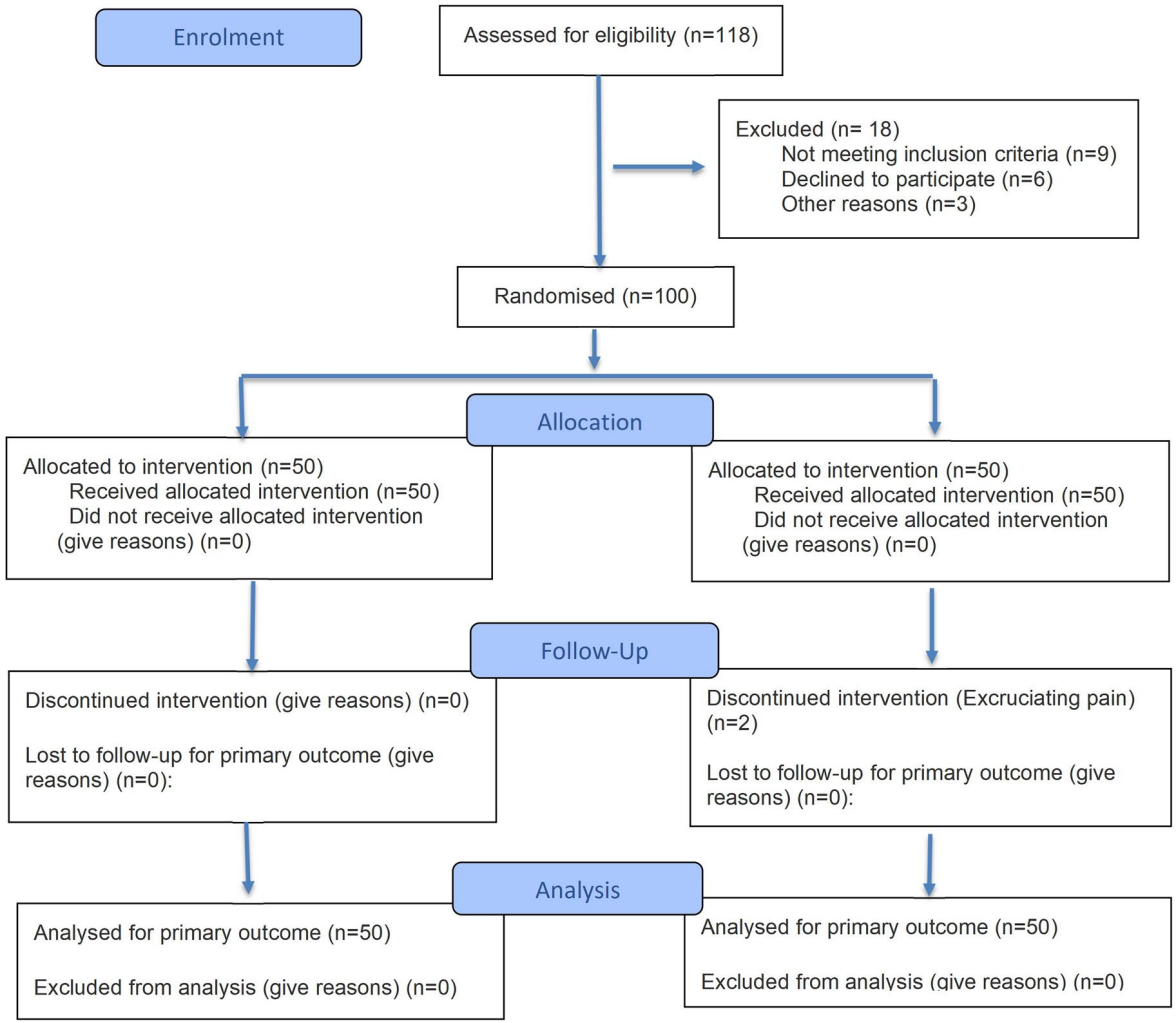
CONSORT flow diagram.

This study was a randomized, double-blind, controlled trial conducted at a Grade A tertiary hospital in eastern China from October 2024 to December 2025. The study was granted ethical approval by the 960th Hospital of the PLA in China (2024-053, 9 May 2024), and the trial was registered with the China Clinical Trial Registry (ChiCTR2400089379) (https://www.chictr.org.cn/showproj.html?proj=236994). All procedures adhered to the ethical standards of the National Research Committee of the 1964 Declaration of Helsinki and its later amendments or comparable ethical standards. Written informed consent was obtained from all participants. The study protocol has been published [19]. This study is reported in accordance with the CONSORT 2025 checklist. Patients requiring rehabilitation after ORIF were eligible to participate in this trial. Participants were recruited and enrolled between November 2024 and October 2025. Individuals who satisfied the predetermined inclusion criteria were recruited into the study: they had experience in performing unilateral ankle ORIF (at least one month post-surgery); they did not have any postoperative complications; they were between 18 and 60 years of age; and they had experienced maximal pain rehabilitation (Visual Analogue Scale (VAS) score ≥4 or greater, indicating a painful experience) during the rehabilitation. Exclusion criteria encompassed the following: contraindications to N_2_O; utilization of the VAS for patients who were unconscious or unable to accurately express pain; and patients who had sustained previous ankle injuries or undergone multiple surgical interventions on the ankle. The CONSORT flowchart for participant enrollment was presented in Figure 1.

### Recruitment

The participants in this study were recruited from a general hospital. A medical investigator was tasked with the responsibility of reviewing the medical records of potential participants in order to identify those who met the stipulated inclusion and exclusion criteria. Following the provision of informed consent, patients were enrolled in the study. Concurrently, the patient’s confidentiality was safeguarded. Patients who elected to participate in the study were provided with two gases free of charge, in addition to treatment and evaluation by rehabilitation professionals.

### Randomization, allocation concealment, and blinding

The randomization process was executed by a statistician using Microsoft Excel. Block randomization was used with a block size of 4. Participants were randomly assigned to two groups at a ratio of 1:1. The personal identifiable information of the patients, which was replaced by a numerical number, was retained by administrators who were not involved in the study. The allocation process was to be conducted with strict confidentiality throughout the study, and the list was to be securely stored in an envelope in a designated room to ensure concealment. Once a participant had met all the necessary enrolment criteria, the appropriate treatment was then assigned to the patient. It was imperative to acknowledge the potential impact of these gases on the outcomes of the experiment. To this end, N_2_O and O_2_ were stored in two cylinders that were indistinguishable in appearance and bore identical white labels, respectively. The dispensing of the gases was unknown to any investigator, other operator, or patient except for the project administrator responsible for dispensing, who was tasked with ensuring that the principle of blinding was practiced. In exceptional circumstances, such as the occurrence of a serious adverse effect, emergency unblinding may be deemed necessary.

### Procedure

The rehabilitation programme commences in the fourth to sixth week following surgery. The programme primarily concentrated on manual therapy session. Manual therapy primarily involves passive ankle joint movements, including passive range of motion exercises such as plantar flexion, dorsiflexion, inversion, and eversion. Each session lasted approximately 20 to 30 min and was conducted by a specialized rehabilitation therapist. Both gases were stored in identical, white-labeled cylinders. A demand-valve system was employed, with gas flow triggered by the patient pressing a button on the device assembly, thereby allowing for intermittent, patient-controlled inhalation on demand. Patients were instructed to press the button with each inspiration only when they experienced pain during manual therapy. A trained nurse was present throughout the session to provide assistance as needed. Considering the time required for N_2_O to take effect, administration was initiated 2 min prior to the commencement of manual therapy during the trial. Manual therapy began once the patient reached an optimal state. To allow the analgesic effect to become established, patients were instructed to begin inhaling two minutes prior to the commencement of manual therapy. They continued to self-administer the gas as needed throughout the entire 20 to 30-minute manual therapy session. The mask was removed immediately upon completion of the session. Total gas exposure ranged from approximately 22 to 32 min; however, the exact number and timing of individual inhalations varied according to each patient’s self-controlled demand. The same delivery method and instructions were applied to both the N_2_O and O_2_ groups to maintain blinding.

The collection of patient demographics and baseline data was conducted 5 min before the treatment (T0). Pain intensity was assessed as a primary indicator using the VAS (a participant self-assessment scale, 0 = no pain, 10 = worst pain imaginable) at 5 min after the beginning of the intervention (T1), 10 min after the beginning of the intervention (T2), and 5 min after the treatment finished (T3) [14,20]. Anxiety levels were measured using a numeric rating scale (NRS) for anxiety (self-report, 0 = no anxiety, 10 = the most intense anxiety one can imagine) at T0 and T3 [21]. Vital signs (heart rate, blood pressure, blood oxygen saturation) were meticulously recorded at T1, T2 and T3, and patients underwent observation for any adverse effects and alterations in their state of consciousness [16,22]. The satisfaction of patients and therapists was measured at T3 [20,23]. Furthermore, patients were questioned on their intentions and willingness to undergo the aforementioned treatment once more, employing analgesic methods at T3 [20,23]. Finally, residual pain was defined as any self-reported pain, regardless of intensity, experienced during the night following the rehabilitation session. This was assessed by asking patients the next morning: ‘Did you experience any pain during the night after the rehabilitation session?’ (Yes/No).

### Sample size

Sample size estimation was performed by statisticians using PASS 2021. The pain was considered the primary endpoint indicator for the trial. With the findings of the preliminary study conducted on 20 participants, subjects who satisfied the stipulated inclusion criteria were methodically allocated to either the N_2_O group or the O_2_ group, with a ratio of 1:1, with a pain score of 3.46 (0.72) vs. 5.05 (0.90). To achieve 90% statistical efficacy and a two-sided test, a sample size of 16 was deemed sufficient. However, considering potential patient dropouts and missing data in actual clinical trials, and to enhance the statistical power and robustness of this study’s results, we ultimately decided to include 100 patients (50 per group).

### Data management

It was imperative to note that all data were treated confidentially. All relevant information studies and electronic data were stored in encrypted files. The Data Security Monitoring Board (DSMC) was established at the inception of the programme. The team consisted of two experts in the field of pain and one specialized rehabilitation therapist, two nurses and a senior statistician. The organization assumed responsibility for representing patients’ rights, assessing safety and the validity of the research process. The DSMC was responsible for reconciling all data on a monthly.

### Data analysis

The raw data were entered into Excel spreadsheets and saved, and were subsequently analyzed statistically using SPSS 27.0 software. This analysis was then double-checked. The Kolmogorov–Smirnov test was utilized to ascertain the normality of the observed indicators. Data were described using mean (standard deviation) or median (inter-quartile range). Count data were expressed as frequencies or percentages. The continuous data were analyzed using the *t*-test or Mann–Whitney *U*-test; the rank-sum test was used for patient satisfaction in both groups; the chi-square test was used to analyse the acceptance, incidence of adverse effects, and the presence of residual pain in the two groups. Generalized estimating equations (GEE) were employed to determine the effects of group, time, and their interaction on pain. The constructed model utilized the covariance matrix among repeated observations. *p* < 0.05 was considered statistically significant.

## Results

### Baseline

Of the 118 participants who were screened, 107 consented to participate and 100 eligible participants of these were enrolled in the study. Participants who had been enrolled were included in the study using a modified intention-to-treat analysis (Figure 1). The demographic and clinical characteristics of the participants in both groups were found to be very similar (Table 1).

**Table 1.** Participant demographic and clinical characteristics.

Project	65% N_2_O (*n* = 50)	O_2_ (*n* = 50)	*p*-value
Age (years)	24.0 (23.0; 28.5)	25.5 (23.0; 28.3)	0.400
Marital status			
Single	31 (62%)	34 (68%)	0.835
Married	19 (38%)	16 (32%)	
Nationality			
Han nationality	46 (92%)	50 (100%)	0.117
Minority nationality	4 (8%)	0 (0%)	
Surgery site			
Left ankle	26 (52%)	28 (56%)	0.841
Right ankle	24 (48%)	22 (44%)	
VAS pain score during rehabilitation	7.0 (6.0; 7.5)	7.0 (6.5; 7.5)	0.256
Measures (T0)			
VAS pain score	0.0 (0.0; 0.0)	0.0 (0.0; 0.0)	1.000
NRS anxiety score	5.0 (3.0; 7.0)	4.0 (3.0; 6.0)	0.338
Heart rate (bpm/min)	80.78 (5.377)	79.16 (7.020)	0.198
Systolic blood pressure (mmHg)	130.50 (6.625)	128.78 (7.147)	0.215
Diastolic blood pressure (mmHg)	81.90 (6.011)	80.06 (6.099)	0.132
Oxygen saturation (%)	99 (99; 99)	99 (99; 99)	0.070

Results are expressed as mean (standard deviation) for normal distribution; median (IQR) for non-normal distribution; and *n* (%) for rank information.

VAS: visual analog scale; NRS: Numerical Rating Scale; T0 = 5 min before the treatment.

### Primary outcome

**Figure 2.**
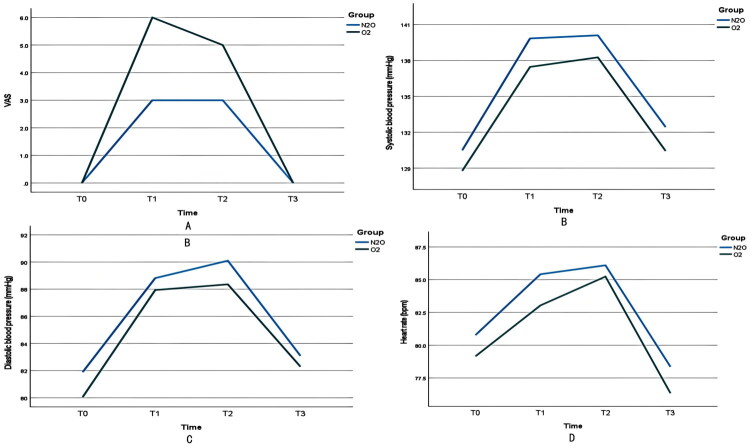
Changes in outcome variable scores. (A) Pain. (B) Systolic blood pressure. (C) Diastolic blood pressure. (D) Heart rate.

The median pain scores of patients in the N_2_O group were significantly lower than those in the O_2_ group at the T1 and T2 time point, and the difference was statistically significant (3.0 (2.0; 3.6) versus 6.0 (5.0; 6.0), median difference −3.0, [95% CI −3.4 to −2.6]; *p* < 0.001; 3.0 (2.0; 3.0) vs. 5.0 (5.0; 6.0), median difference −2.0, [95% CI −2.8 to −1.2]; *p* < 0.001). However, at the T3 time point, no statistical difference was observed between the two groups (0.0 (0.0; 0.0) versus 0.0 (0.0; 0.0), median difference 0.0, [95% CI 0.0 to 0.0]; *p* = 0.41). Furthermore, in terms of the time effect, no statistically significant difference was observed between the T1 and T2 time points in either the intervention group or the control group (*p* = 0.10) (Table 2 and Figure 2A). The MCID obtained by different methods were SRM = 0.888, ES = 1.415 and SEM = 0.847, respectively.

**Table 2.** GEE of the differences between groups for pain score.

Group	T0	T1	T2	T3	*p* (T0 versus T1)	*p* (T0 versus T2)	*p* (T0 versus T3)	*p* (T1 versus T2)	*p* (T1 versus T3)	*p* (T2 versus T3)
65% N_2_O	0 (0.0; 0.0)	3.0 (2.0; 3.6)	3.0 (2.0; 3.0)	0.0 (0.0; 0.0)	<**0.001**	<**0.001**	0.737	1.000	<**0.001**	<**0.001**
O_2_	0 (0.0; 0.0)	6.0 (5.0; 6.0)	5.0 (5.0; 6.0)	0.0 (0.0; 0.0)	<**0.001**	<**0.001**	0.083	0.211	<**0.001**	<**0.001**
Median difference (95% CI)	0 (0 to 0)	−3.0 (−3.4 to −2.6)	−2.0 (−2.8 to −1.2)	0 (0 to 0)						
*p*	1.000	<**0.001**	<**0.001**	0.413						

Data are median (Q1; Q3). N_2_O: nitrous oxide; O_2_: oxygen. Bold type indicates significant differences.

T0 = 5 min before the treatment.

T1 = 5 min after the beginning of the intervention.

T2 = 10 min after the beginning of the intervention.

T3 = 5 min after the treatment finished.

### Secondary outcomes

#### Anxiety

The results of the anxiety level are also given in Table 3. The median anxiety scores (Q1; Q3) of the two groups did not demonstrate a significant difference at T3 (0.0 (0.0; 1.0) versus 0.0 (0.0; 2.0), median difference 1.0, [95% CI −0.8 to 0.8]; *p* = 0.31, *η*^2^ = 0.027) (Table 3).

**Table 3.** Vital signs and anxiety score.

			95% CI		Median difference/Mean difference (95% CI)	Student’s *t*-test		Effect size
Variable	65% N_2_O (*n* = 50)	O_2_ (*n* = 50)	Lower	Upper		*t* value	*p*-value	Cohen’s *d*
Heart rate (bpm)								
T1	85.42 (9.804)	83.04 (6.955)	82.54	85.92	2.38 (−0.99 to −5.75)	1.400	0.165	0.280
T2	86.10 (11.079)	85.24 (6.346)	83.89	87.45	0.86 (−2.72 to 4.44)	0.476	0.635	0.095
T3	78.36 (6.960)	76.34 (5.531)	76.09	78.61	2.02 (−0.48 to 4.52)	1.607	0.111	0.321
Systolic blood pressure (mmHg)								
T1	139.84 (9.325)	137.46 (8.377)	136.88	140.42	2.38 (−1.14 to 5.90)	1.343	0.183	0.269
T2	140.10 (8.572)	138.26 (8.475)	137.49	140.87	1.84 (−1.54 to 5.22)	1.079	0.283	0.216
T3	132.44 (6.309)	130.44 (6.165)	130.19	132.69	2.00 (−0.48 to 4.48)	1.603	0.112	0.321
Diastolic blood pressure (mmHg)								
T1	88.82 (5.695)	87.94 (6.702)	87.15	89.61	0.88 (−1.59 to 3.35)	0.708	0.481	0.142
T2	90.10 (7.357)	88.36 (7.131)	87.79	90.67	1.74 (−1.14 to 4.62)	1.201	0.233	0.240
T3	83.10 (6.628)	82.30 (5.737)	81.47	83.93	0.80 (−1.67 to 3.26)	0.645	0.520	0.129

**Table:** 

						Mann–Whitney *U*		
						*Z* value	*p*-value	Eta squared (*η*^2^)
Oxygen saturation (%)								
T1	99 (98; 99)	99 (99; 100)	99	99	0 (0 to 0)	−3.613	<**0.001**	0.100
T2	99 (99; 99)	99 (99; 99)	99	99	0 (0 to 0)	−2.286	**0.022**	0.052
T3	99 (99; 99)	99 (99; 99)	99	99	0 (0 to 0)	−0.902	0.367	0.009
Anxiety scores								
T3	0.0 (0.0; 1.0)	0.0 (0.0; 2.0)	0.0	1.0	1.0 (−0.8 to 0.8)	−1.013	0.311	0.027

Data are mean (standard deviation) and median (Q1; Q3). N_2_O: nitrous oxide; O_2_: oxygen. Bold type indicates significant differences.

T1 = 5 min after the beginning of the intervention. T2 = 10 min after the beginning of the intervention. T3 = 5 min after the treatment finished.

#### Vital signs

The vital signs of the two groups are displayed in Table 3. No statistically significant differences were observed in heart rate and blood pressure at T1, T2 and T3 between the two groups. However, a statistically significant difference in oxygen saturation was observed at T1 and T2 (T1: 99 (98; 99) versus 99 (99; 100), median difference 0, [95% CI 0 to 0]; *p* < 0.001; T2: 99 (99; 99) versus 99 (99; 99), median difference 0, [95% CI 0 to 0]; *p* = 0.02) (Figure 2B–D). Repeated measures ANOVA revealed significant main effects of time for heart rate, systolic blood pressure, and diastolic blood pressure (*p* < 0.001), indicating that vital signs significantly increased during the intervention period (T1, T2) and significantly decreased post-treatment (T3) across all participants. However, the group * time interaction was not significant for any of the three measures (Heart rate: *p* = 0.73; systolic blood pressure: *p* = 0.95; diastolic blood pressure: *p* = 0.65), indicating parallel trends in vital sign changes between the 65% N_2_O and O_2_ groups without significant differences. Additionally, the main effects between groups were not significant (*p* > 0.05), suggesting no statistically significant differences in overall vital sign levels between the two groups (Table 4).

**Table 4.** Univariate analysis of vital signs.

Variable	Group	T0	T1	T2	T3	Main effect of group	Group × time interaction	Main effect of time
Heart rate (bpm)								
	65% N_2_O	80.78 (5.377)	85.42 (9.804)	86.10 (11.079)	78.36 (6.960)	2.211	0.323	**44.668**
	O_2_	79.16 (7.020)	83.04 (6.955)	85.24 (6.346)	76.34 (5.531)	*p* = 0.140	*p* = 0.733	***p* = 0.000**
Systolic blood pressure (mmHg)								
	65% N_2_O	130.50 (6.625)	139.84 (9.325)	140.10 (8.572)	132.44 (6.309)	2.155	0.113	**131.254**
	O_2_	128.78 (7.147)	137.46 (8.377)	138.26 (8.475)	130.44 (6.165)	*p* = 0.145	*p* = 0.952	***p* = 0.000**
Diastolic blood pressure (mmHg)								
	65% N_2_O	81.90 (6.011)	88.82 (5.695)	90.10 (7.357)	83.10 (6.628)	1.404	0.529	**116.949**
	O_2_	80.06 (6.099)	87.94 (6.702)	88.36 (7.131)	82.30 (5.737)	*p* = 0.239	*p* = 0.650	***p* = 0.000**

Data are mean (standard deviation). N_2_O: nitrous oxide; O_2_: oxygen. Bold type indicates significant differences.

T0 = 5 min before the treatment.

T1 = 5 min after the beginning of the intervention.

T2 = 10 min after the beginning of the intervention.

T3 = 5 min after the treatment finished.

#### Satisfaction

Within the N_2_O group, 40 patients (80%) expressed a high level of satisfaction with N_2_O, 9 patients (17%) indicated a moderate level of satisfaction, and 1 patient (2%) conveyed a low level of satisfaction. In the O_2_ group, 5 (10%) patients were satisfied with oxygen, 21 (42%) expressed uncertainty, and 21 (42%) were dissatisfied (*p* < 0.001). A statistical discrepancy was identified between the two groups with regard to satisfaction among rehabilitation therapists (*p* < 0.001) (Table 5).

**Table 5.** Satisfaction of patients and doctors.

Variable	65% N_2_O (*n* = 50)	O_2_ (*n* = 50)	Mann–Whitney *U*	
			*Z* value	*p*-value
Patients satisfaction (%)				
Very dissatisfied	0(0)	3(6)	−8.591	<**0.001**
Dissatisfied	1(2)	21(42)		
Uncertainty	0(0)	21(42)		
Satisfied	9(18)	5(10)		
Very satisfied	40(80)	0(0)		
Doctors satisfaction (%)				
Very dissatisfied	0(0)	3(6)	−9.132	
Dissatisfied	0(0)	25(50)		
Uncertainty	1(2)	17(34)		<**0.001**
Satisfied	1(2)	5(10)		
Very satisfied	48(96)	0(0)		

N_2_O: nitrous oxide; O_2_: oxygen. Bold type indicates significant differences.

#### Acceptance, adverse effects and residual pain

The level of acceptance was found to be significantly higher in participants receiving N_2_O than in those receiving O_2_. Furthermore, 49 (98%) participants in the N_2_O group were willing to use the same gas in future. Throughout the intervention, no severe adverse effects related to gas inhalation were observed. Within the N_2_O group, participants experiencing mild adverse effects, including dizziness (5 patients) and nausea (2 patients), typically did not necessitate treatment. Residual pain was observed in 3 (6%) of the N_2_O group and 13 (26%) of the O_2_ group, which was statistically significant in both groups (*p* = 0.01) (Table 6).

**Table 6.** Other secondary outcomes.

Variable	65% N_2_O (*n* = 50)	O_2_ (*n* = 50)	Chi-square test	
			*χ* ^2^	*p*-value
Acceptance (%)				
Yes	49(98)	8(16)	68.58	<**0.001**
No	1(2)	42(84)		
Side effects (%)				
Yes	7(14)	0(0)	7.527	**0.012**
No	43(86)	50(100)		
Residual pain (%)				
Yes	3(6)	13(26)	6.027	**0.012**
No	47(94)	37(74)		

N_2_O: nitrous oxide; O_2_: oxygen. Bold type indicates significant differences.

## Discussion

The present randomized controlled trial evaluated the short-term analgesic effect of inhaled N_2_O in patients with acute pain during postoperative rehabilitation following ankle fractures. The present study demonstrated that the administration of 65% N_2_O resulted in superior analgesia, higher satisfaction levels, and a mild adverse effects when compared with the O_2_.

This study found that after inhaling 65% N_2_O, the median pain score of patients at the midpoint decreased from 7.0 points at baseline to 3.0 points. In contrast, the control group only showed a decrease from 7.0 points to 6.0/5.0 points, resulting in a median difference between groups of −2.0 to −3.0 points. This analgesic effect aligns closely with the findings of Wang et al. [20] regarding the rehabilitation of anterior cruciate ligament reconstruction surgery. In their study, the VAS score in the N_2_O group decreased from approximately 6.5 points at baseline to about 3.5 points, yielding an intergroup difference of −3.0 points (95% CI: −4.0 to −2.0) when compared to the placebo group. The effect sizes from both studies are comparable, indicating that N_2_O exhibits stable and reproducible analgesic effects in acute pain management during orthopedic postoperative rehabilitation. Furthermore, Delafontaine et al. [24] reported that N_2_O (Kalinox) provided effective pain relief during postoperative physical therapy in patients with cerebral palsy, demonstrating a significant difference compared to placebo (*p* < 0.01). However, not all orthopedic or rehabilitation scenarios have reached similar conclusions. For instance, Uglow [25] found that in closed reduction surgery for anterior shoulder dislocation, a 50% concentration of N_2_O did not demonstrate any additional analgesic advantage compared to intravenous opioids, with no statistically significant difference in the VAS between the groups (*p* > 0.05). Similarly, Dupeyron et al. [26] did not observe any improvement in pain relief with N_2_O during intensified therapy for adhesive capsulitis of the shoulder joint. This discrepancy may arise from variations in the nature and mechanisms of pain. This study specifically addresses mechanical and motion-induced pain during postoperative rehabilitation, which is closely associated with peripheral and central sensitization following tissue injury, as well as traction stimulation of adherent soft tissues during joint activity [27]. N_2_O exerts a favorable regulatory effect on this type of pain by activating the descending noradrenergic pathway and inhibiting spinal glutamate transmission [28–30]. In contrast, shoulder joint diseases frequently involve intricate central and peripheral mixed pain mechanisms [27]. Furthermore, the duration of closed reduction operations is typically very brief, which may not adequately demonstrate the benefits of patient-controlled administration of N_2_O. Additionally, variations in administration methods and dosages may influence efficacy. Our preliminary research indicates that the duration of N_2_O inhalation has little effect on the degree of pain relief, with pain scores remaining similar at five and ten minutes post-inhalation. This may be related to nitrous oxide’s inherent analgesic characteristics: rapid onset and rapid dissipation of its pain-relieving effects [14]. However, we have not identified an optimal time point that achieves adequate analgesia while minimizing gas wastage, a topic we hope to explore further in future studies.

Anxiety scores did not differ significantly between groups (*p* = 0.31, *η*^2^ = 0.027), consistent with the findings reported by Straube et al. [31] N_2_O did not significantly reduce anxiety levels in patients undergoing *in vitro* fetal head inversion (*p* > 0.05). However, several studies have demonstrated significant anxiolytic effects of N_2_O in acute procedural settings [32,33]. The lack of a significant effect in our study may be attributed to several factors. First, our patients’ baseline anxiety levels were relatively low, leaving little room for further reduction. Unlike one-time operations, postoperative patients with ankle fractures typically undergo 4–6 weeks of observation before recovery, which may lead to a greater familiarity with the treatment environment and medical staff, potentially reducing procedure-related anxiety. Second, previous reports have indicated a correlation between pain and anxiety [34]. While N_2_O significantly reduced pain in our study, the corresponding reduction in anxiety did not reach statistical significance. This suggests that pain relief alone may not fully account for the anxiolytic effects of N_2_O. Therefore, the improvement in anxiety may depend not only on pain relief but also on psychological factors, such as patients’ expectations regarding the rehabilitation process and their adaptation to the treatment environment, as well as the placebo effect of the inhalation device itself. Future studies should measure anxiety at multiple time points, including before and during rehabilitation sessions, to more accurately capture the anxiolytic effects of N_2_O.

Our data indicate that although both patient groups exhibited significant changes in heart rate, systolic blood pressure, and diastolic blood pressure over time during treatment, neither the main effects between groups nor the group * time interactions were significant. This implies that inhalation of 65% N_2_O did not induce heart rate or blood pressure change patterns distinct from those observed in the O_2_ group. Regarding oxygen saturation, although statistically significant differences were observed at T1 and T2 time points, the median values were 99% in both groups with extremely small absolute differences, suggesting potentially limited clinical significance. Wang et al. reported that in patients undergoing rehabilitation after anterior cruciate ligament reconstruction, inhalation of 65% N_2_O was associated with stable vital signs, showing no significant differences between the N_2_O and oxygen groups [20]. Similarly, a pediatric study found no significant changes in vital signs during procedural pain management with premixed N_2_O [35]. Bindu et al. [36] indicated that in patients undergoing cerebellopontine tumor surgery under sevoflurane anesthesia, N_2_O did not result in significant between-group differences in heart rate or blood pressure; notably, the control group exhibited higher incidences of intraoperative tachycardia and hypotension. Our protocol of 20–30 min of intermittent exposure falls within this transient window, and the absence of significant between-group differences suggests that the mild cardiovascular stimulation induced by nitrous oxide was insufficient to produce a distinct hemodynamic signature compared to oxygen alone. 14% of patients in the 65% N_2_O group reported side effects (primarily transient, known nitrous oxide effects like dizziness and nausea), while none were reported in the O_2_ group. However, considering these effects were typically transient and mild, with no serious adverse events, and given the significant analgesic benefits, we deem the safety profile of 65% N_2_O acceptable. N_2_O can be administered by trained and qualified nurses in clinics or triage rooms, several studies have reported that the use of this gas is safe in clinical settings [37,38].

Up to 98% of patients in the 65% N_2_O group expressed willingness to use this regimen again for similar future treatments, compared to only 16% in the O_2_ group. Similarly, patient and physician satisfaction scores were significantly higher in the 65% N_2_O group than in the O_2_ group. Moreover, residual pain the following morning was reported by 6% of the N_2_O group versus 26% of the O_2_ group (*p* = 0.012), indicating that better pain control during rehabilitation may reduce post-session pain. These consistent findings demonstrate that 65% N_2_O not only effectively relieves pain but also delivers a treatment experience more readily accepted by both patients and operating physicians. This is crucial for enhancing the quality of orthopedic outpatient or postoperative rehabilitation training. Therefore, N_2_O appears to be a safe alternative to other medications for managing acute pain in patients undergoing postoperative rehabilitation training for ankle fractures.

This study also has limitations. First, this study was conducted in a single centre. The majority of participants were male, although this was not intentional. This phenomenon can be attributed to the fact that the majority of fractures are caused by exercise and are therefore more prevalent among young men [3]. It is imperative that the results of the study be confirmed in a multi-center setting, as well as in both male and female subjects. Second, the investigation did not encompass the evaluation of short- or long-term functional indicators. Notably, we did not measure active range of motion (AROM) during or immediately after the rehabilitation session, which is a direct outcome of physical therapy that may be influenced by pain control. Additionally, we did not assess longer-term functional endpoints. Future studies should include serial assessments of short- or long-term functional indicators to determine whether the analgesic benefits of nitrous oxide translate into objectively improved joint mobility and enhanced efficacy of physical therapy. Third, transient side effects occurred exclusively in the nitrous oxide group, potentially leading to partial unblinding of certain participants or personnel. Future studies should formally evaluate the success of blinding. Fourth, the primary outcome relied on patient self-reporting. Pain perception and tolerance can vary significantly among individuals, which may introduce reporting bias. While randomization aims to evenly distribute such individual differences across groups, we cannot entirely dismiss the impact of subjective variability. All these factors should be taken into account in future research.

## Conclusion

The present study revealed a substantial discrepancy in pain levels among subjects who were randomly assigned to receive either N_2_O or O_2_. Patient and rehabilitation therapist satisfaction and acceptance of N_2_O was higher than that of O_2_, and adverse effects were mild. However, further studies are required to evaluate the long-term results in the early postoperative period, with the aim of determining whether this results in improvements. It is imperative that this finding is corroborated and expanded upon in subsequent studies.

## Data Availability

The data sets used and/or analyzed during the current study are available from the corresponding author on reasonable request and with appropriate institutional and ethics approval.

## Open scholarship

This article has earned the Center for Open Science badge for Preregistered. The materials are openly accessible at https://www.chictr.org.cn/showproj.html?proj=236994.
